# Role of Surgery in the Multimodal Treatment of Pituitary Carcinoma: A Retrospective Single-Institution Case Series

**DOI:** 10.3390/cancers18132064

**Published:** 2026-06-25

**Authors:** Christina Abi Faraj, Maxwell Tran, Sherise D. Ferguson, Maria A. Gubbiotti, Heather Y. Lin, Dima Suki, Nazanin Majd, Steven G. Waguespack, Ian E. McCutcheon

**Affiliations:** 1 Department of Neurosurgery, M D Anderson Cancer Center, Houston, TX 77030, USA; cafaraj@mdanderson.org (C.A.F.);; 2 Department of Radiation Oncology, Holzer Center for Cancer Care, Gallipolis, OH 45631, USA; 3 Department of Pathology, M D Anderson Cancer Center, Houston, TX 77030, USA; 4 Department of Biostatistics, M D Anderson Cancer Center, Houston, TX 77030, USA; 5 Department of Neuro-Oncology, M D Anderson Cancer Center, Houston, TX 77030, USA; 6 Department of Endocrine Neoplasia and Hormonal Disorders, M D Anderson Cancer Center, Houston, TX 77030, USA

**Keywords:** pituitary carcinoma, metastasis, surgery, pituitary adenoma, pituitary neuroendocrine tumor

## Abstract

Introduction: Pituitary carcinoma (PC) is a rare, aggressive endocrine neoplasm characterized by metastasis and challenging clinical management. The transformation from pituitary adenoma (PA) to PC is poorly understood, and predictors of metastasis remain elusive. This study evaluates the clinical course, surgical outcomes, and molecular characteristics of PC. Methods: We retrospectively reviewed patients with PC treated at the M. D. Anderson Cancer Center between 1993 and 2023. Primary outcomes included metastasis-free survival and overall survival (OS). Clinical features, radiographic findings, surgical strategies and outcomes, immunohistochemical profiles, and MIB-1 were analyzed. Results: The cohort (*n* = 20) had a median age at PA and PC diagnosis of 33.9 and 43.3 years, respectively. The median metastasis-free interval was 7.4 years. GH- and ACTH-secreting tumors showed shorter times to PC diagnosis, while nonfunctioning PAs had longer metastasis-free survival. PAs with MIB-1 > 10% had shorter survival. Dura was the most common site of metastasis within the CNS, and bone was the most common outside the CNS. Leptomeningeal disease was seen in six patients. PAs became aggressive > five years after initial surgical resection (*n* = 13) or metastasized early within the first five years (*n* = 7). Median OS from PA diagnosis was 13.7 years, and 8.6 years from PC diagnosis. A total of 102 neurosurgical procedures were performed, with a median of five per patient; the median was similar in patients surviving longer than five years vs. those whose survival was shorter (5.0 vs. 4.5 procedures, *p* = 0.661). Most surgical interventions post-PC diagnosis were for optic decompression or metastasectomy. All long-term survivors (at least five years after PC diagnosis) received temozolomide-based therapy, with most also receiving radiotherapy. Conclusions: PC shows a variable clinical course, with some PAs progressing to PC after years, while others transform rapidly. All long-term survivors received temozolomide-based therapy, most in combination with radiotherapy and repeated surgical intervention, suggesting that aggressive multimodal management may be associated with prolonged survival. Future research will focus on identifying reliable predictors of metastasis at different time points in the complex clinical evolution of these tumors.

## Simple Summary

Pituitary carcinoma (PC) is an exceptionally rare and aggressive type of brain tumor that originates from the pituitary gland and spreads to other parts of the brain or body. Because so few cases exist worldwide, little is known about how best to manage it surgically. This study presents the experience of a single major cancer center with 20 patients treated over three decades—the largest such surgical series reported to date. We describe the full course of treatment these patients underwent, including the number and types of operations performed, the outcomes of those operations, and the complications that arose. We found that PC shows a variable clinical course, with some adenomas progressing to PC after years, while others transform rapidly. All long-term survivors received temozolomide-based therapy, most in combination with radiotherapy and repeated surgical intervention, suggesting that aggressive multimodal management may be associated with prolonged survival; however, given the retrospective design and small sample size, these findings should be interpreted as hypothesis-generating and require validation in larger prospective studies. Our findings aim to guide surgeons and patients in making more informed treatment decisions for this challenging disease.

## 1. Introduction

Pituitary carcinomas (PCs) make up only 0.1–0.5% of all pituitary tumors [1,2,3,4,5,6,7,8]. They are defined by the presence of systemic or craniospinal metastases distant from the primary pituitary tumor [9]. According to current World Health Organization (WHO) guidelines, no specific histologic or molecular markers are required to diagnose PC [9]. Among pathologists, an effort has arisen to describe such tumors as metastatic pituitary neuroendocrine tumors (PitNETs), but in this study, we retain their established identity as “pituitary carcinomas.” Such tumors often (but not invariably) share histological characteristics including invasive growth, increased mitotic index, and significant expression of mutant p53 protein [5,7].

Currently, neither the malignant transformation of a pituitary tumor nor the clinical course of an established PC can be reliably predicted, and the rarity of this disease continues to impede the identification of prognostic markers and optimal treatment strategies. It remains controversial whether PCs arise in a stepwise fashion, beginning as a benign pituitary adenoma and gradually developing malignant characteristics [1,10,11,12,13], or whether they can arise de novo [1,14,15]. The extent of metastatic spread is variable, ranging from a single lesion to widespread metastatic disease. These tumors can disseminate either by hematogenous or by craniospinal spread. Patients develop cranio-spinal (40–45%) or systemic metastasis (39–47%), while 13–16% exhibit both [16,17]. Prolactin-secreting PCs are the most common subtype, followed by those secreting ACTH.

Historically, PCs are associated with a poor prognosis, with 66% of patients surviving less than one year from metastasis diagnosis, and a median overall survival of approximately 31 months [18,19,20]. Patients with systemic metastases have the shortest survival [21].

The clinical presentation of PC is highly variable and depends on the functional state of the tumor, as well as the location and size of any metastatic lesions [22]. The diagnosis of metastases is most often preceded by the development of new symptoms or hormonal changes. Patients most commonly present with symptoms attributable to mass effect. However, in some cases, metastases are asymptomatic and discovered accidentally on surveillance imaging or postmortem. The 2025 Revised European Society of Endocrinology Guidelines recommend diagnosing an aggressive pituitary tumor in any patient with an invasive pituitary tumor demonstrating unusually rapid progression or clinically relevant tumor progression despite optimal standard therapies, including surgery, radiotherapy, and conventional medical treatment [23]. They recommend screening for metastatic disease in patients with aggressive pituitary tumors when site-specific symptoms arise, when biochemical and radiological findings are discordant, or before initiating chemotherapy, and should include at a minimum brain and spine MRI along with a whole-body evaluation method such as FDG-PET or DOTATOC-PET. The definitive diagnosis of PC can only be established upon pathologic confirmation of a metastatic lesion discontinuous from the primary sellar tumor, either within the craniospinal axis or systemically. Early identification is of major clinical importance, as these tumors are associated with significantly increased morbidity and mortality even prior to the development of overt metastases [23].

The challenges of diagnosing and managing PCs are well documented [2,18,24,25]. The literature reports a variable latency period from the initial diagnosis of a pituitary tumor to evidence of metastasis, with a mean of 6.6 years (range, 3 months–18 years) [21]. The principal difficulty lies in formulating a reliable set of criteria to predict which pituitary adenomas will behave aggressively and eventually metastasize. Although their timely identification and treatment might prevent metastasis, that is often difficult to achieve given the rarity of this disease. PCs typically demonstrate histological features such as anaplasia, elevated mitotic count, and necrosis, which are congruent with their clinical aggressiveness (i.e., multiple recurrences, resistance to treatment). However, tumors histologically defined as atypical in prior classification schemes can be clinically benign [26,27,28]. This dilemma has led to increased interest in identifying genetic mutations and biological markers that predict clinically aggressive behavior [26,29]. Quantitative analysis of Ki-67 labeling index and p53 expression by immunohistochemistry (IHC) are useful tests that correlate with the degree of invasiveness in PAs. However, there are no pathognomonic IHC or molecular markers to predict malignant behavior [20,22].

In addition to their diagnostic challenges, PCs typically require complex multidisciplinary management that has defied standardization [5,6,30,31]. This includes surgical resection (often multiple), radiation therapy (limited-field or stereotactic radiosurgery), and chemotherapy. The European Society of Endocrinology (ESE) endorsed temozolomide (TMZ) monotherapy as the primary adjuvant treatment for PCs [23,32]. Recent reports have described the use of targeted therapies (e.g., VEGF and mTOR inhibitors) and immunotherapy (e.g., pembrolizumab), but the data are limited [13,33,34,35,36,37,38,39,40,41]. Despite multimodal therapy, many patients die from significant disease progression. Because of the rarity of this disease, much of the extant knowledge on its management and long-term outcomes comes from case reports and small case series. Specifically, information on the surgical management of this disease is limited. Here we present our clinical series of 20 patients with PC with an emphasis on surgical management and outcomes of these patients. This is the largest single-institution clinical series of PCs described in the literature, and the only one that focuses on the surgical treatment of this tumor type.

## 2. Methods

### 2.1. Study Population

This retrospective study was conducted under an institutional-review-board-approved protocol. Twenty consecutive patients with the diagnosis of pituitary carcinoma were identified in The University of Texas M.D. Anderson Cancer Center (MDACC) Department of Neurosurgery database between 1993 and 2023. All patients had a history of primary pituitary tumor with documented systemic (extracranial) or central nervous system (CNS) metastases. The diagnosis of pituitary carcinoma, or metastatic PitNET, required the presence of a metastatic lesion systemically or in the form of discontinuous tumor spread within the CNS, according to the current WHO guidelines [9]. This criterion has remained the sole defining feature of pituitary carcinoma across all WHO classification editions relevant to our study period, including the 2004, 2017, and 2022 classifications. While the 2017 and 2022 WHO updates introduced lineage-based transcription factor subtyping and revised tumor nomenclature (replacing “pituitary adenoma” with “pituitary neuroendocrine tumor”), neither update altered the fundamental diagnostic threshold for malignancy. As such, patients diagnosed with atypical or aggressive pituitary adenomas without evidence of a distant metastatic lesion were excluded, as were patients with other known cancer primaries whose metastatic lesions could not be confirmed to be of pituitary origin.

Patients with active disease or undergoing post-treatment surveillance were followed with MRI of the brain and relevant systemic sites every 3–6 months, while clinically stable patients were followed at 6- to 12-month intervals.

Variables collected included patient demographics, date of diagnosis of PA and PC, metastasis location, pathologic features including immunohistochemical and (when available) transcription factor staining for the sellar tumor and its corresponding metastasis, visual outcomes following surgery, neurosurgical procedures and their corresponding indications and complications, systemic and radiation therapies, radiographically proven progression following treatment for the sellar and/or metastatic CNS tumor, and date of death or last follow-up.

### 2.2. Neuropathological Studies

All tumors were evaluated by a board-certified neuropathologist. H&E-stained sections were reviewed, and histological features were documented. Immunohistochemical stains were performed to assess pituitary transcription factors, pituitary hormones, and mitotic index (MIB-1). Such immunohistochemical stains allowed for subtyping of the pituitary tumors based on hormonal expression and/or lineage.

### 2.3. Statistical Analysis

Descriptive statistics (frequency distribution, mean [±standard deviation], and median [range]) were used to summarize patient characteristics. To characterize the healthcare burden of a PC diagnosis on patients, we calculated the frequency of administered treatments before and after the diagnosis of PC. The frequency before PC diagnosis was obtained by dividing the total number of treatments the patient received between PA and PC diagnosis by the time interval from PA to PC diagnosis. To obtain the frequency of administered treatments after the diagnosis of PC, the total number of treatments the patient received from PC diagnosis until death was divided by the time interval from PC diagnosis until death.

The chi-square test or Fisher’s exact test was used to test differences in category variables, and the Wilcoxon rank-sum test or Kruskal–Wallis test was used to detect differences for continuous variables between the two groups. The Kaplan–Meier method was used for the analyses of overall survival (OS) and time to PC diagnosis. OS was defined as the time from the date of PA or PC diagnosis until death. For events that had not occurred by the time of data analysis, times were censored at the last time the patient was known to be alive. A log-rank test was performed to gauge the difference in survival between groups. *p*-Values of <0.05 were considered statistically significant. Analyses were conducted using GraphPad Prism 10.0.3 (GraphPad Prism Software, New York, NY, USA). Given the small sample size of this cohort, all subgroup analyses, including those examining the relationship between hormonal subtype, MIB-1 labeling index, metastasis localization, radiotherapy, temozolomide-based therapy, and the number of neurosurgical procedures with survival outcomes, should be considered exploratory and hypothesis-generating. These analyses were not powered to detect statistically significant differences, and their results should be interpreted with appropriate caution.

## 3. Results

### 3.1. Patient and Tumor Characteristics

**Figure 1:**
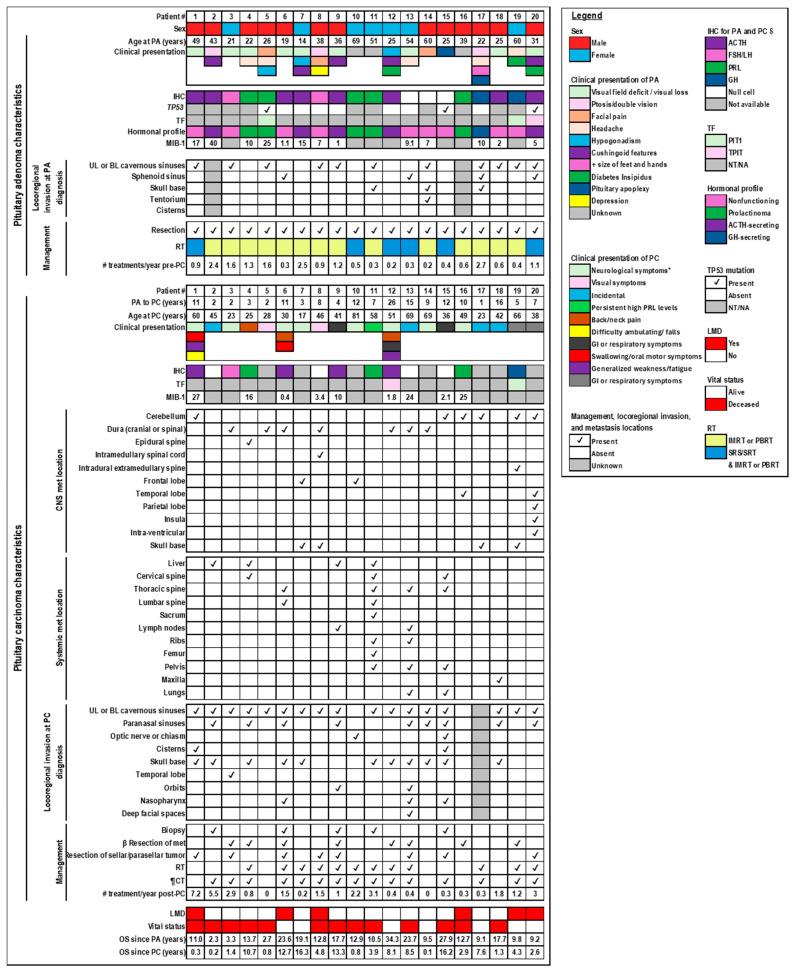
Oncoprint summary table of the 20 patients with pituitary carcinoma. ⸹ PA specimen in patient 20 showed diffuse ACTH consistent with densely granulated subtype; he developed Crooke cell adenoma at recurrence. Mutational analysis of the recurrent suprasellar tumor revealed high tumor mutational burden (236 mut/Mb) and multiple somatic mutations, including *MSH2/MSH6* and *ATRX*. ¶ Patient 5 received only one dose of TMZ post-PC diagnosis. # treatments/year includes all surgeries, radiation therapy, and systemic therapy regimens the patient received, divided by the time interval from PA to PC or from PC to death or last follow-up. * Neurological symptoms include headache, nausea/vomiting, altered mental status, dysarthria/aphasia, sensory symptoms, and seizures. β Patient 9 underwent percutaneous ablation of hepatic metastasis. Abbreviations: ACTH, adrenocorticotrophic hormone; BL, bilateral; CT, chemotherapy; FSH, follicle-stimulating hormone; GH, growth hormone; IHC, immunohistochemistry; LMD, leptomeningeal disease; LH, luteinizing hormone; met, metastasis; NT/NA: Not tested or not available; OS, overall survival; PA, pituitary adenoma; PC, pituitary carcinoma; PIT-1: pituitary-specific transcription factor 1; PRL, prolactin; RT, radiotherapy; TF: transcription factors; TMZ, temozolomide; TPIT: T-box transcription factor.

**Figure 2:**
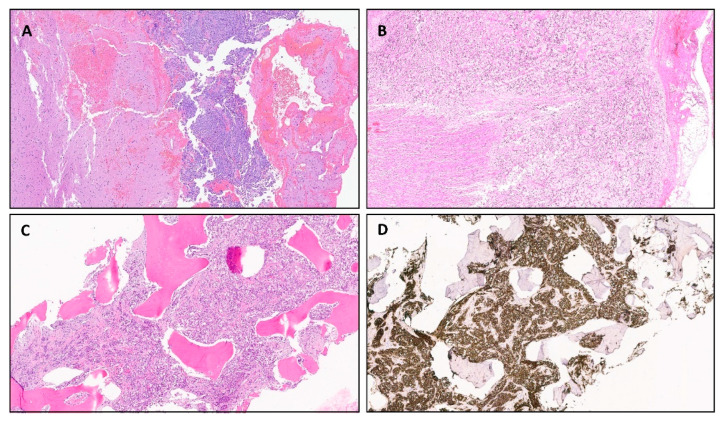
Histologic examples of metastatic pituitary neuroendocrine tumors/carcinoma. (**A**) Metastasis to the temporal lobe showing the metastatic tumor composed of cells with eosinophilic cytoplasm and medium-to-large, round-to-ovoid nuclei arranged around vessels, imparting a vaguely papillary appearance. The adjacent involved brain parenchyma is noted. (**B**) Nests of metastatic tumor involving the optic nerve (bottom left) with periorbital adipose tissue noted at the periphery. (**C**) Bone involved by metastatic tumor showing nests and clusters of cells with pale to clear cytoplasm. There is a somewhat fibrotic background between clusters of tumor cells. (**D**) Prolactin immunohistochemical stain showing diffuse and strong cytoplasmic expression within the metastatic tumor. This immunohistochemical profile is similar to that of the primary tumor.

Twenty patients were included in this study, of whom 65% were males (Table 1, Figure 1). The median age at primary pituitary tumor diagnosis was 33.9 (range, 14.3–69.3) years. Immunohistochemical staining for pituitary hormones was available for all primary tumors, with a plurality of staining for ACTH (40%) and prolactin (25%). Nine patients had non-functional adenomas (three silent corticotroph, three null cell, one silent somatotroph adenoma, and two positive for FSH and/or LH), five had ACTH-secreting tumors, five had prolactinomas, and one had a GH-secreting tumor. No tumors were TSH-secreting or plurihormonal. One patient’s diagnosis was updated as Crooke cell adenoma at recurrence, given the tumor’s high mitotic and proliferative activity and positive staining for CAM5.2 cytoplasmic rings. This tumor exhibited a high mutational burden, including *MSH2/MSH6* and *ATRX* mutations. Transcription factor analyses indicated PIT1-lineage in two patients and TPIT-lineage tumor in one. MIB-1 was available for 12 patients, and the median MIB-1 was 9.6% (range, 1.1–40.0), with high MIB-1 (>3%) present in most primary sellar tumor samples (10/12, 83%). The highest MIB-1 was present in ACTH+ (*n* = 6, median 10%, range 1.1–40%), PRL+ (*n* = 2, 10 and 24.8%), and GH+ (*n* = 1, 10%) tumors. Functioning PAs had higher MIB-1 (median 12.5%, range 5–40%) than non-functioning ones (median 7.1%, range 1.1–17.2%). Immunohistochemical data and proliferation indices were unavailable for the remaining patients. Histological examples of the metastases are shown in Figure 2.

Excluding three patients with incomplete data, all were symptomatic at initial presentation, with visual problems the most common symptom (*n* = 12, 60%). At PA diagnosis, 65% (*n* = 13) of patients showed locoregional invasion, most commonly into one cavernous sinus (*n* = 8, 62%). Five patients (25%) had invasion into both cavernous sinuses, and four (20%) into the sphenoid sinus. Six patients (30%) had extension into other neighboring areas such as the clivus, superior orbital fissure, Meckel’s cave, and more laterally placed bone structures. Patients with locoregional invasion at the time of initial surgery for PA were more likely to require subsequent resection or adjuvant radiation therapy. For instance, patient 17 underwent frontotemporal craniotomy, re-do transsphenoidal surgery, and proton beam radiotherapy for residual cavernous sinus tumor. At PC diagnosis, all patients (except for one patient with incomplete data) had locoregional invasion. Finally, seven patients (35%) presented with microadenomas at initial PA diagnosis (patients 2, 9, 10, 12, 17, 18, and 20), of whom five harbored ACTH-staining tumors, one had a prolactinoma, and one had a GH-secreting tumor.

### 3.2. Patient and PC Tumor Characteristics

**Figure 3:**
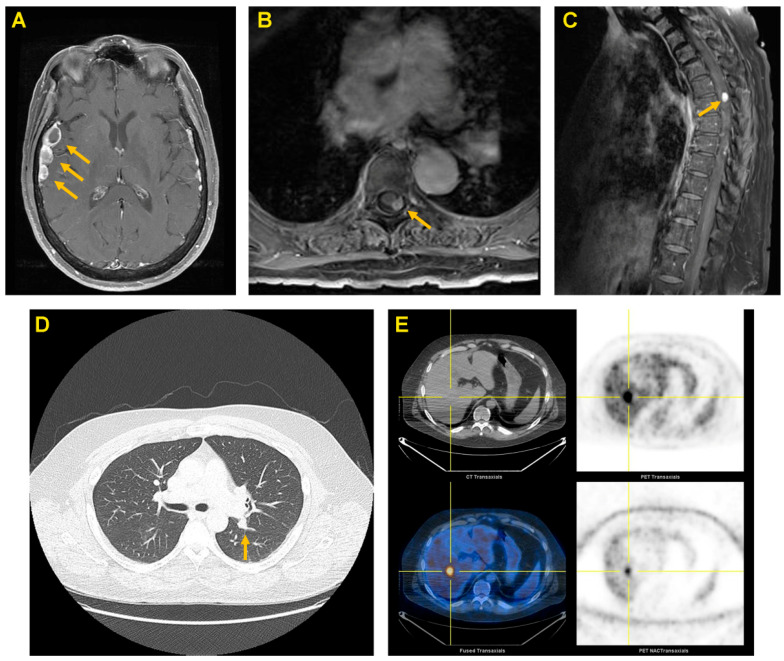
Pathology-proven metastases from pituitary carcinoma to other central nervous system (right temporal dura, spine) and systemic (lungs, liver) locations. (**A**) T1-weighted post-contrast MRI in the axial plane showing multifocal right temporal dural metastases in patient 12. (**B**,**C**) T1-weighted fat-saturated post-contrast MRI in the axial (**B**) and sagittal (**C**) plane showing an intradural extramedullary nodule at the T6 level causing asymptomatic cord flattening in patient 19. (**D**) CT of the chest showing a 1.2 cm nodule (arrow) posterior to the left main pulmonary artery in patient 15. (**E**) PET/CT showing pituitary carcinoma metastasis in the right lobe of the liver in patient 9.

The median age at PC diagnosis was 43.3 years (range, 17.1–81.4). Most metastases were confined to the CNS (*n* = 12, 60%). CNS locations of metastasis included dura (*n =* 10), most commonly the temporal dura (*n* = 5). Eight of these 10 patients had dural metastasis as the only CNS metastasis. Additional CNS locations for metastasis included cerebellum (*n* = 3), skull base (*n* = 2), and spinal cord or intradural extramedullary spine (*n* = 2), and individual instances of temporal, parietal, insula, and intraventricular metastasis. Of all patients with systemic involvement (*n* = 8, 40%), four patients (50%) had isolated systemic metastases. Bone was the most common systemic site (*n* = 6, 75%), followed by liver (*n* = 4, 50%), lungs (*n* = 2, 25%), and cervical lymph nodes (*n* = 2, 25%). Patients with both CNS and systemic metastases had non-functioning PA (*n* = 3) or prolactinoma (*n* = 1). Figure 3 depicts metastatic lesions in various locations.

Hormonal status of the metastatic lesion was available for 13 patients (65%). The hormonal profile of the metastatic sample matched that of the primary tumor in all cases except for patients 2 and 8, whose primary tumors stained for ACTH and FSH/LH, respectively, while the corresponding metastatic lesions did not stain for any hormones. MIB-1 in metastatic lesions ranged from 0.4% to 30%. Mutational analyses of the metastasis revealed *TP53* mutations in patient 15 and *MSH6* mutations in patient 16, who also had an unmethylated *MGMT* promoter. Immunostaining for transcription factors on resected metastases showed positivity for TPIT in case 12 and PIT-1 in case 19.

At PC diagnosis, most patients (*n* = 14, 70%) were symptomatic, with headache the most common complaint (*n* = 4). Other symptoms included new-onset seizures, neck, back, or abdominal pain, new neurological deficits, and confusion. Metastasis was incidental in 20% (*n* = 4), detected through imaging for other complaints (*n* = 3) or during intraoperative LN dissection during the resection of a highly invasive sellar tumor (*n* = 1; patient 13).

In three patients, the metastasis was initially thought to be a meningioma, and a craniotomy was performed to prove its metastatic nature. Patient 12 had dural-based nodules that had been observed for almost ten years on the assumption that they were meningiomas. A craniotomy was later done because the lesions were growing, increasing in number, and becoming cystic. Similarly, patient 3 had a temporal dural metastasis, removed three months after its detection due to a significant increase in tumor size. In patient 15, a dural nodule on the posterior fossa convexity was followed for seven years, after which the patient presented with a cough that triggered chest imaging showing metastases in the lungs and thoracic vertebrae.

### 3.3. Neurosurgical Management of PA and PC

**Figure 4:**
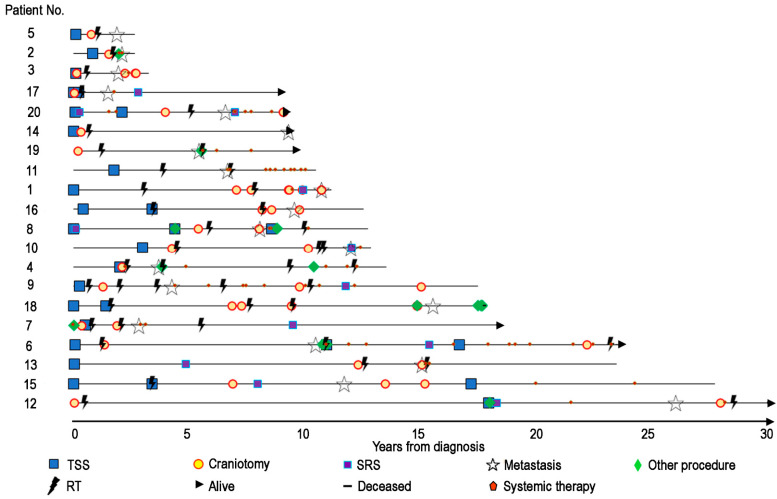
Treatment regimens for all 20 patients, sorted by length of survival. Systemic therapy includes: Conventional chemotherapy (e.g., carboplatin, vincristine, fluorouracil, temozolomide, cisplatin, etoposide, doxorubicin, lomustine, capecitabine) and targeted therapy (e.g., bevacizumab, pembrolizumab, infigratinib, denosumab, tipifarnib, and sorafenib; or procedures related to the disease, but not directly for tumor resection, such as bilateral adrenalectomy); Other procedures include: ventriculoperitoneal shunt placement, removal, or revision, external ventricular drain placement, lumbar drain placement, biopsy of sellar mass, and any spinal surgery such as laminectomy and fusion. Abbreviations: RT: radiation therapy; SRS: stereotactic radiosurgery; TSS: transsphenoidal surgery.

Figure 4 provides a treatment timeline of our twenty PC patients from initial PA diagnosis and shows highly variable courses. Some followed an aggressive course from the outset (e.g., patients 2–5, 7, 9, and 17), while others initially had a benign course and subsequently developed metastasis (e.g., patients 1, 6, 8, 10–16 and 18–20). Management of PC included all three forms of multimodal therapy (chemotherapy, radiation therapy, and surgical resection) in nine patients. Following PC diagnosis, the frequency of neurosurgical procedures increased from 0.6 to 0.9 per year. Similarly, treatment frequency (neurosurgical procedures, chemotherapy, and radiotherapy) increased from one per year to 1.7 per year. These numbers exclude non-neurosurgical procedures such as bilateral adrenalectomy and biopsy or resection of systemic metastases. Of the 20 patients, metastatic disease was histologically confirmed in 13 patients, either through surgical resection or biopsy of the metastatic lesion. In the remaining seven patients (patients 5, 7, 10, 14, 17, 18, 20), the diagnosis of metastasis was established on radiographic grounds, based on the presence of a new lesion discontinuous from the primary sellar tumor on MRI or cross-sectional imaging, in the appropriate clinical context of a known aggressive pituitary tumor with hormonal or radiographic progression. Systemic metastases were more likely to be biopsied, while CNS metastases were more likely to be surgically resected.

Over the course of the disease, 102 neurosurgical procedures were performed, with a median of five procedures per patient (range 1–12). This count excludes non-neurosurgical procedures, such as adrenalectomies or biopsies of lung or liver nodules. Nearly all patients (95%, *n* = 19) underwent a transsphenoidal resection or biopsy of their primary pituitary lesion, with a total of 28 transsphenoidal procedures performed, of which 43% (*n* = 12) were for optic pathway decompression. In 16 patients (64%), the first procedure was a transsphenoidal operation for PA resection. Six patients underwent craniotomies for residual disease after an initial transsphenoidal surgery. Two patients (12 and 19) underwent an upfront craniotomy. A total of 41 craniotomies were performed, with vision preservation being the primary goal in 34% (*n* = 14) of cases. Fourteen patients (70%) had an initial subtotal resection, while the extent of resection in others was unknown. A total of 27 craniotomies and eight TSSs were performed for sellar tumor recurrence; one was a fronto-orbitozygomatic and another a modified orbito-zygomatic craniotomy. One patient had a combined transsphenoidal and bifrontal craniotomy, and another had a trans-facial/lateral rhinotomy approach. Half of the cohort required one or more craniotomies for tumor recurrence. Of the 69 intracranial tumor resections performed, gross total resection was achieved in five procedures (7%).

Fourteen patients underwent at least one neurosurgical procedure after the diagnosis of PC. One patient (patient 6) had six procedures performed after PC was discovered. Procedures were often for optic apparatus decompression, metastasectomy, or to mitigate complications of the disease or of prior surgery (ventriculoperitoneal shunt for hydrocephalus, hematoma evacuation, lumbar drain for CSF leak, SRS for metastasis or sellar tumor recurrence). Procedures included biopsies of liver, lung, sacrum, or T8 vertebra; one patient had a hepatic lobectomy, one patient had percutaneous ablation of his liver metastases, and six patients underwent surgical resection of the metastatic tumor (three involving the spine and three involving the temporal dura). Of the five patients with ACTH-secreting tumors, four underwent bilateral adrenalectomies for uncontrolled hypercortisolism. This was performed prior to PC diagnosis in two patients and after PC diagnosis in two patients. None of these four developed Nelson’s syndrome.

### 3.4. Radiation Therapy

Radiation therapy was administered to all patients before PC diagnosis, with 11 receiving adjuvant radiation after their first PA resection (patients 2–4, 6, 8, 9, 11, 12, 14, 17, and 20). Radiation typically included intensity-modulated radiation therapy (IMRT) to 45–54 Gy in 25–30 fractions, or single- or multi-fraction stereotactic radiosurgery (SRS). Overall, eighteen SRS sessions were conducted in ten patients over the course of their disease (one fraction in eight patients; five fractions in two patients). SRS was performed as adjuvant treatment following surgical resection of the primary sellar tumor (*n* = 4), sellar tumor recurrence (*n* = 3), or treatment for the metastatic lesions (*n* = 5). After PC diagnosis, radiation therapy targeted the sellar/parasellar areas (*n* = 3), with metastases treated by IMRT (*n* = 6), SRS (*n* = 5), and craniospinal (*n* = 2) or proton beam (*n* = 1) irradiation.

### 3.5. Chemotherapy

Sixteen patients received chemotherapy treatments over the course of their disease. These included conventional chemotherapy and other non-conventional therapies such as immune checkpoint inhibitors (pembrolizumab) and targeted therapies (bevacizumab, infigratinib, denosumab, tipifarnib, and sorafenib). Two patients received adjuvant chemotherapy following their first PA resection (patients 4 and 20). Six received chemotherapy for invasive parasellar disease prior to PC diagnosis, while 14 received chemotherapy after PC diagnosis (one patient received only one dose of TMZ). Patient 7 received up-front chemotherapy with carboplatin/vincristine prior to any surgical resection following an initial biopsy of the sellar tumor that revealed low-grade glioma. On later review, that diagnosis was revised to pituitary adenoma.

Upon PC diagnosis, chemotherapy was offered upfront, alone or in combination with radiation therapy, in nine patients. The most common systemic therapy regimen was TMZ monotherapy (*n* = 11, 55%), followed by capecitabine/TMZ (*n* = 6, 30%), bevacizumab (*n* = 5, 25%), pembrolizumab (*n* = 3, 15%), and cisplatin/etoposide (*n* = 2, 10%). One patient (11) received multiple chemotherapeutic regimens including cisplatin and etoposide, denosumab, TMZ monotherapy, capecitabine and TMZ, pembrolizumab (clinical trial 2015–0948), clinical trial combination drugs (2016-0529: FAZ053: anti-PD-L1 IgG4 antibody, PDR001: anti-PD-1 antibody), clinical trial combination drugs (2012-0061: bevacizumab, temsirolimus and valproic acid), and octreotide; all these regimens failed to halt disease progression and the patient expired four months after cessation of treatment later.

Four patients did not receive chemotherapy or radiation therapy after PC diagnosis, with two undergoing surgical resection of the primary sellar/parasellar tumor or of the metastasis. Of those two, one survived less than a year, and the other nearly three years. A third patient was lost to follow-up after one month, and the fourth underwent placement of a ventriculoperitoneal shunt and an external ventricular drain and expired one year after PC diagnosis.

### 3.6. Patient Outcomes

*Visual outcomes* (Table 2). Visual deficits improved after 15 of 24 neurosurgical interventions performed to decompress the optic apparatus. Of these, three patients underwent TSS followed by craniotomy for further surgical debulking and had significant improvement in vision or fully regained it. Worse or new visual deficits occurred postoperatively following six procedures, and stable vision was seen following three procedures. Only one patient (4) had complete vision loss as an initial presentation of PA. Overall, patients 4, 5, 7, 9, 10, 18, and 20 exhibited complete vision loss in one or both eyes over the course of their disease.

*Complications* (Table 2, Table 3 and Table 4). Fifteen patients (75%) had postoperative complications for a total of 45 complications reported. The most common complications encountered were CSF leak (*n* = 9, 20%), followed by transient or permanent diabetes insipidus (*n* = 7, 16%), hypo/panhypopituitarism (*n* = 6, 13%) and cranial nerve palsy (*n* = 5, 11%). The two instances of intracranial hemorrhage occurred after PC diagnosis. Complication rates increased with successive procedures (excluding SRS): 16.7% at first surgery, 41.2% at second, 40.0% at third, and 72.7% at fourth or later procedures. Compared to first operations, fourth-or-later procedures carried a significantly higher complication risk (OR = 14.29, *p* = 0.012 by Fisher’s exact test with Bonferroni correction). These complication rates were calculated by examining each sequential procedure across all patients. That is, complication occurrence at the first procedure was assessed across all 20 patients, at the second procedure across those who underwent a second operation, and so forth.

*Leptomeningeal disease* (Figure 1). Six patients (30%) eventually developed leptomeningeal disease (LMD). It was diagnosed concurrently with PC in three patients, two months after PC in one patient, and three years after PC in two patients. Of these six patients, four also had dural-based metastases. Although not all dural-based metastases were associated with LMD, six of ten patients with metastasis to the dura did demonstrate LMD as well.

*Hydrocephalus.* Patients 8 and 18 required surgical management for hydrocephalus (Table 2). Patient 8 developed hydrocephalus as a sequela of LMD, while patient 18 developed hydrocephalus twice, initially from the sellar tumor blocking both foramina of Monroe and subsequently from shunt malfunction.

*Time from PA to PC*. The median interval from PA to PC diagnosis was 7.4 years (95% Confidence interval [CI]: 2.8–10.8; range, 1.5–26.1). GH-secreting tumors and ACTH-secreting tumors had the shortest time to PC diagnosis (median, 1.5 and 4.3 years, respectively). Patients with nonfunctioning PAs had a longer median survival without metastasis (median, 10.8 years) than those with functioning PAs (median, 4.3 years; *p* = 0.353) (Supplementary Figure S1A). Furthermore, as MIB-1 of the PA increased, the interval between PA and PC diagnosis shortened, and PAs with a MIB-1 of ≥10% at diagnosis had a significantly shorter time to metastasis (median, 2.5 years) compared to PAs with MIB-1 < 10% (median, 10.1 years; *p* = 0.013) (Supplementary Figure S1B). Locoregional invasion at PA diagnosis did not seem to impact significantly the interval between PA and PC (*p* = 0.271) (Supplementary Figure S1C).

*Overall survival*. At the last follow-up, 13 patients were deceased. Median follow-up from PA diagnosis was 23.59 years, and 12.75 years from PC diagnosis. Median OS from PA diagnosis was 13.7 years (95% CI: 11–23.6), while the median OS from PC diagnosis was 8.6 years (95% CI: 1.4–13.3). The one-, two-, and five-year OS rates after metastasis diagnosis were 79% (95% CI: 53–92%), 74% (95% CI: 48–88%), and 51% (95% CI: 26–71%), respectively.

Younger patients (<43.3 years) tended to live longer after PC diagnosis (median 13.4 vs. 3.9 years; *p* = 0.053; Supplementary Figure S2A). PAs with MIB-1 < 10% were associated with longer survival (median 8.6 vs. 5.7 years for MIB-1 ≥ 10%; *p* = 0.72; Supplementary Figure S2B). Patients with both CNS and systemic metastases showed better survival than those with only CNS or systemic spread alone (median 13.4 vs. 4.8 vs. 3.0 years; *p* = 0.378; Supplementary Figure S2C). The time between PA and PC diagnosis did not significantly impact survival (median 10.7 vs. 4.8 years for intervals > 7.4 vs. ≤7.4 years; *p* = 0.637). Functioning PAs were linked to longer survival after PC diagnosis (median 10.6 vs. 4.8 years for nonfunctioning tumors; *p* = 0.848). None of these differences reached statistical significance.

All long-term survivors, defined as survival > five years after PC diagnosis, were treated with TMZ-based therapy, and all but one long-term survivor received radiotherapy to the primary tumor or its metastasis. Patients receiving any radiotherapy post-PC diagnosis (median, 10.7 vs. 2.5 years; *p* = 0.112; Supplementary Figure S2D) and those receiving TMZ-based therapy (median, 10.7 vs. 1.1 years; *p* < 0.0001; Supplementary Figure S2E) both had improved median OS from PC diagnosis. There is a slight positive association between the number of neurosurgical procedures and OS since PA diagnosis, but the correlation appears weak (Supplementary Figure S3).

## 4. Discussion

Pituitary carcinoma is a rare and formidable disease, with limited data available on its optimal surgical management and outcomes. Treating patients with pituitary carcinoma is often complex, typically necessitating multiple surgical resections to control the tumors and alleviate symptoms. Despite a comprehensive, multidisciplinary approach involving surgery, radiation therapy, and chemotherapy, patient outcomes remain poor. It is still unknown whether pituitary carcinomas develop de novo or transform from previous pituitary adenomas, making it difficult to predict which patients will experience an aggressive clinical course and metastasis. In this study, we present our experience with twenty patients with PC.

### 4.1. Course of Transformation to Pituitary Carcinoma

PCs exhibit a highly variable course, with latency periods ranging from very short to very long following the diagnosis of the original PAs. While many PCs tend to follow an aggressive path from the outset, some initially present a clinically benign course before developing aggressive features. It remains unclear whether these changes are due to inherent and spontaneous alterations in a malignant tumor or whether the tumor gradually progresses to develop aggressive characteristics over time [5,6,7].

In our study, the median time from PA to PC was 7.4 years, aligning with other studies in the literature [3,42]. Importantly, we show four different presentations of PC: a clinically aggressive PA course and early metastasis (patients 7, 9), a clinically aggressive PA course and late metastasis (patients 1, 8, 10, 13, 15, 16, 18, 20), a clinically benign PA course and early metastasis (patients 2, 3, 4, 5, 17), and a clinically benign PA course and late metastasis (patients 6, 11, 12, 14, 19). A clinically aggressive PA course was defined by ≥2 disease recurrences that have been treated with radiation or surgery prior to metastasis diagnosis. An early metastasis presentation was defined as ≤5 years from PA diagnosis, with a late presentation being >5 years from PA diagnosis. Thus, early metastasis does not necessarily signify a more aggressive PA. Some benign tumors may accumulate genetic aberrations leading to aggressive behavior and early metastasis [14,43]. Conversely, some PAs may have inherent metastatic potential from the start without significant local progression as a prerequisite.

For example, patients 6, 12, and 13 experienced long latency periods with relatively stable PA courses before metastasis detection, whereas patients 1 and 18 had PAs that exhibited aggressive behavior with multiple recurrences despite optimal management. Since not all aggressive pituitary tumors transform to become PC, it will be crucial going forward to identify and characterize predictors of metastasis in this patient population, as well as molecular differences between the sellar tumor and its metastatic progeny. We did note changes in the immunohistochemical profile between the original sellar tumor and the metastatic lesion in our study.

Some metastases, such as those to the dura, can remain stable (or grow very slowly) for many years. In our series, two patients had dural metastases initially misdiagnosed as meningiomas, and one patient with a solitary dural-based cerebellar convexity metastasis was followed for years with relative stability. Thus, a tumor need not always demonstrate aggressive growth before it metastasizes. It remains unclear what clinicopathologic differences exist between patients who die soon after PC diagnosis and those who survive with more stable disease. Therefore, a high index of suspicion for metastatic disease should be maintained regardless of the apparent clinical behavior of the primary tumor, as dural lesions in patients with a known history of pituitary adenoma should not be presumptively attributed to meningioma without pathologic confirmation.

### 4.2. Predictors of Transformation to Pituitary Carcinoma

Predicting which pituitary lesions will progress to carcinoma remains challenging [44]. Potential scenarios that suggest aggressive pituitary tumor include invasive corticotroph macroadenomas in men, macro-/giant prolactinomas and somatotroph invasive macroadenomas resistant to medical treatment, invasive nonfunctioning macroadenomas switching to functioning tumors, rapid progression following surgical resection, progression following radiation therapy, or tumors with a high Ki67 index (>10%), high mitotic count, extensive mutant *TP53* expression, or *TP53* and/or *ATRX* mutations [5].

PCs are more likely to express ACTH and prolactin than are benign pituitary tumors. The proportion of silent corticotroph tumors among PCs is also higher than in benign PAs. As PAs evolve into PCs, a switch from nonfunctioning to functioning tumors may be observed, indicating that nonfunctioning tumors are less likely to metastasize or to demonstrate aggressive potential [3,5,6,7,45]. However, data are mixed on the significance of functional status in the development of aggressive phenotypes [46,47].

In our patients, functioning PAs developed metastasis earlier than their nonfunctioning counterparts. Eighty percent of patients with ACTH-secreting tumors required bilateral adrenalectomy, a significantly higher rate than the 7–18% reported in other studies, underscoring the aggressive and difficult-to-manage nature of metastatic corticotroph tumors [48,49]. These findings highlight the complexity of correlating functional status with aggressive behavior and the need for further investigation into the role of functional status in predicting PC development.

Proliferative markers such as MIB-1 have been suggested as predictors of aggressive behavior, with elevated levels associated with invasive properties and an increased risk of local recurrence in PAs [28,50,51,52]. However, the role of MIB-1 in predicting later metastasis remains uncertain [18,25,53]. Our study found that primary PAs with an MIB-1 ≥ 10% had shorter metastasis-free survival. Traditionally, studies analyzing outcomes of PAs have used a threshold of 3–5%, above which residual tumors are more likely to progress. However, in our cohort, the median PA MIB-1 was 9.6%, with only two cases having a MIB-1 < 3%. This suggests that a 3% cutoff is inadequate.

PAs with an MIB-1 ≥ 10% are typically more aggressive and invasive, but a low MIB-1 does not exclude aggressive tumors nor the future onset of metastasis [5]. Our data indicate that patients with a MIB-1 ≥ 10% may be considered for more vigilant surveillance; however, given the incomplete availability of MIB-1 data across the cohort, this finding should be considered exploratory. The question of whether this surveillance should include both cranial and systemic imaging remains unresolved and is difficult to address in a retrospective study. Additionally, we found that a lower MIB-1 in the original sellar tumor was associated with longer median survival (8.6 vs. 5.7 years), indicating a potential link between MIB-1 in the PA and the behavior of the metastatic tumor derived from it.

Our cohort included three patients whose PAs harbored *TP53* mutation, known to be associated with aggressive tumor subtypes and frequently found in aggressive pituitary tumors and PCs [5,6,50,54]. Corticotroph tumors with *ATRX* mutations also tend to be more aggressive [55]. One patient in our study developed Crooke cell adenoma at PA recurrence before metastasis detection. This patient had already received TMZ for PA management. The acquisition of hypermutation and somatic *MSH2*/*MSH6* mutations following alkylating chemotherapy (e.g., TMZ) has been reported in other cancer types (e.g., *MGMT* promoter-hypermethylated diffuse gliomas and neuroendocrine tumors), so similar mechanisms might be at play in PCs [56,57]. Crooke’s cell adenoma is classified as an aggressive variant of PA [43], and a high index of suspicion for PC should be given to patients with Crooke cell adenoma presenting with a lesion elsewhere in the craniospinal axis or systemically. Although we aimed to include more biological markers in our analysis, inconsistent testing in our cohort limited the statistical robustness of our findings. Furthermore, transcription factor staining and molecular testing, including *TP53*, *ATRX*, and mismatch repair analyses, were available only in a minority of patients, precluding any systematic conclusions regarding molecular predictors of malignant transformation.

Two-thirds of PAs in our study exhibited locoregional invasion at presentation, which some authors have associated with aggressive behavior [22,58]. These tumors are challenging to manage due to the increased likelihood of subtotal resection and the need for multiple operations to achieve reasonable tumor control, as illustrated in our case series. However, our findings indicate that locoregional invasion at presentation does not significantly affect time to metastasis; this is consistent with the fact that most locally aggressive tumors do not consistently have metastatic potential and with the rarity of PCs in all large series of tumors of pituitary origin. All cases showed locoregional invasion at the time of PC diagnosis, with a highly variable latency from PA to metastasis.

### 4.3. Management of Pituitary Carcinoma: The Role of Surgery

Over the course of their disease from PA diagnosis until last follow-up, patients with PC typically require a complex multidisciplinary management approach, including multiple surgeries. Our study is the first to examine the surgical nuances and outcomes in patients with PC. Medical treatments for patients with aggressive pituitary tumors are often ineffective in controlling tumor growth and corresponding compressive symptoms. Therefore, repeated surgery was done in most of our cohort and may be considered in patients with aggressive or recurrent pituitary tumors or PCs, particularly when visual disturbances necessitate optic apparatus decompression [59,60,61,62,63]. In fact, a survey by the ESE on the management of aggressive pituitary tumors and PCs showed that 77.4% of aggressive PAs and 80.5% of PCs had been treated at least twice using surgery, with 27.9% being surgically treated four times [3]. However, remission rates after repeated transsphenoidal surgery are significantly lower [59,60,61,62,63].

In our study, half of our patients underwent five or more neurosurgical procedures over the course of their disease, with one patient undergoing 12 procedures. Following PC diagnosis, half of our patients required one or more interventions. We observed an increase in the frequency of any treatment administration from one treatment/year to 1.7 treatments/year on average after metastasis detection. Similarly, the number of surgeries/year increased from 0.6/year to 0.9/year. Prior to metastasis detection, PAs often required repeat surgeries, including transcranial approaches, particularly for tumors extending into the suprasellar or lateral parasellar regions. Extended transsphenoidal approaches may also be considered based on the surgeon’s experience and on tumor extent [64].

Surgical interventions following PC diagnosis addressed various issues, including optic apparatus decompression, management of complications (e.g., hydrocephalus, shunt malfunction, hemorrhage), and removal of metastatic disease within the CNS. Furthermore, most surgical resections were subtotal. Despite the extensive surgical efforts, achieving total or near-total resection is rare for aggressive pituitary tumors due to their infiltrative nature and propensity for rapid recurrence [65]. Therefore, the potential palliative benefits of subtotal resection in patients with PC should be weighed against the potential risks [66,67]. Repeat procedures are less successful over time and carry an increasing risk of complications (e.g., CSF leak, hemorrhage, worsened visual fields, optic nerve palsy, hypopituitarism, and diabetes insipidus) due to the anatomical complexity associated with post-surgical scarring [68]. These surgeries can (and indeed, must) be combined with other treatments, such as systemic and/or radiological therapies, to achieve better tumor control.

Our data suggest that aggressive multimodal treatment can lead to prolonged survival. We observed a longer patient survival from the diagnosis of metastasis to the last follow-up than previously reported in the literature [42]. Repeat surgical resections of recurrent metastases may prolong survival [6,21,48]. Complete surgical excision of isolated metastases, followed by adjuvant radiotherapy, gives diagnostic confirmation of metastasis and may be associated with prolonged disease control and improved quality of life in selected patients [69,70,71]. In our cohort, repeated surgical intervention served primarily diagnostic, decompressive, cytoreductive, and palliative roles, as well as addressing complications of disease or prior surgery and enabling resection of selected accessible metastases. While a positive association was observed between the number of neurosurgical procedures and overall survival, this may reflect survival bias—patients who lived longer simply had more opportunity to undergo repeated interventions—rather than a causal relationship between surgical burden and improved survival.

In addition to repeat resection, the use of TMZ in our patients with PC was associated with improved OS, and other authors who incorporated TMZ into chemotherapy treatment for PC showed similar survival rates of around 50% at 5 years [33,72]. It is important to acknowledge that this observation is subject to survival bias—patients who survived longer had a greater opportunity to receive temozolomide, radiotherapy, and repeated surgical interventions, making it impossible to establish a causal relationship between any specific treatment and prolonged survival from this retrospective dataset alone. Prospective studies and multi-institutional registries will be essential to disentangle the effects of treatment from the inherent biological variability of this disease.

### 4.4. Limitations of This Study

This study has several limitations. First, given the retrospective design and small sample size, these findings should be interpreted as hypothesis-generating and require validation in larger prospective multi-institutional registries. The retrospective design also subjects the manuscript to selection bias. The sample size precludes conducting multivariate analyses to identify independent predictors of shorter time to metastasis and shorter overall survival or to compare the effectiveness of specific treatment modalities. Even in the univariate analyses of these two endpoints, we reported only statistical test results, i.e., *p*-values, for comparisons with a relatively reasonable sample size and number of events in each group. All subgroup and survival analyses in this study are exploratory in nature, given the small cohort size, and no definitive conclusions regarding the prognostic or therapeutic significance of any individual variable can be drawn from these data alone.

As a retrospective series spanning 30 years at a major referral center, imaging and clinical visit frequency varied across the cohort depending on disease activity, clinical status, and the degree of involvement of referring institutions. The lack of a standardized follow-up imaging protocol represents an inherent limitation of the study design. Further, over the study period, substantial advances occurred across all domains relevant to the management of PC, including neurosurgical techniques (expanded endoscopic approaches, intraoperative imaging, and neuronavigation), imaging quality and resolution, radiation therapy delivery (transition from conventional fractionated radiotherapy to stereotactic radiosurgery and intensity-modulated techniques), systemic therapy (introduction and adoption of temozolomide, targeted agents, and immune checkpoint inhibitors), and pathological classification. These changes introduce significant heterogeneity in the diagnostic workup, treatment strategies, and surveillance protocols applied across the cohort, limiting direct comparisons between patients treated in earlier versus later decades and precluding definitive conclusions about the relative contribution of any specific intervention to outcomes.

The sequential, heterogeneous, and individualized nature of the therapeutic regimens administered further limits the attribution of outcomes to any single intervention. Underestimation of postoperative complications is also a possibility due to incomplete records from outside institutions. The complication analysis does not account for the non-independence of repeated observations within the same patient. Furthermore, patients reaching fourth or later procedures represent a more aggressive and surgically complex subgroup with greater disease burden and more extensive prior surgical scarring, factors that independently contribute to higher complication rates. Accordingly, the observed increase in complication rates with successive procedures should be interpreted descriptively rather than as a causal relationship between procedural number and complication risk.

Mutational and transcription factor analyses were not available for many of our patients due to inadequate sample availability, lack of surgical resection of metastatic lesions, or resection performed at an outside institution. These limitations hinder direct comparisons and conclusions regarding molecular predictors of metastasis development. Furthermore, the diagnosis of metastatic disease was histologically confirmed in 65% of our patients; in the remainder, the diagnosis was established radiographically. This represents an inherent limitation of retrospective surgical series in rare diseases, where tissue confirmation of every lesion is not always clinically feasible or warranted.

## 5. Conclusions

PC is a rare and challenging malignancy that requires complex multidisciplinary treatment consisting of surgical resection, medical therapy, and radiotherapy. Prolonged survival following the detection of metastasis was observed in our cohort with this approach, particularly when incorporating TMZ-based therapy, though prospective validation is needed. Patients typically require multiple surgical resections for tumor and symptom control, and these procedures carry an elevated risk of postoperative complications. This disease course is highly variable with latencies between PA and PC of varying duration, suggesting potential intrinsic biological differences beyond those that trigger metastasis. Low MIB-1 does not eliminate the risk of transformation of PA to PC, though tumors with MIB-1 < 10% may be associated with better outcomes. Future studies are needed to identify reliable predictors of metastasis and compare pituitary tumors that exhibit aggressive behavior prior to metastasis with those that do not.

## Figures and Tables

**Table 1:** Patient and tumor characteristics.

Variable		All Patients, *N* = 20	%
Age at PA diagnosis (years)	Median (range)	33.9 (14.3–69.3)	
Gender	Female	7	35
	Male	13	65
Clinical presentation at PA diagnosis ^α^	Visual problems	12	60
	Headache	5	25
	Cushing’s	5	25
	Other (facial pain, depression)	4	20
	Hypogonadism	3	15
	Diabetes insipidus	3	15
	Apoplexy	2	5
	Acromegaly	1	5
PA IHC staining	ACTH	8	40
	PRL	5	25
	GH	2	10
	Null cell	3	15
	LH	1	5
	FSH, LH	1	5
PA hormonal profile	ACTH-secreting	5	25
	Nonfunctioning	5	25
	Prolactin-secreting	5	25
	GH-secreting	1	5
	Silent corticotroph	3	15
	Silent somatotroph	1	5
PA MIB-1 ^α^	Median (range)	9.6 (1.1–40.0)	
Locoregional invasion at PA diagnosis ^α^		13	65
Age at PC diagnosis (years)	Median (range)	43.3 (17.1–81.4)	
Interval between PA and PC (years)	Median (range)	7.4 (1.5–26.1)	
Clinical presentation at PC diagnosis ^α^	Incidental	4	20
	Headache	4	20
	Neck/back pain	3	10
	Neurological deficit	2	10
	Swallowing/oral motor symptoms	2	10
	Visual symptoms	2	10
	Generalized weakness/fatigue	2	10
	Persistently elevated PRL levels despite adequate surgical, medical, and radiation treatment	1	5
	GI or respiratory symptoms	1	5
	Confusion	1	5
	New-onset seizure	1	5
	Difficulty ambulating/falls	1	5
PC MIB-1 ^α^	Median (range)	6.7 (0.4–30.0)	
Locoregional invasion at PC diagnosis ^α^		19	95
Metastasis location	CNS only	12	60
	Systemic only	4	20
	CNS and systemic	4	20
Systemic metastasis location	Bone	6	30
	Liver	4	20
	Lung	2	10
	LN	2	10
No. of recurrences prior to PC diagnosis	Median (range)	2 (0–6)	
No. of neurosurgical procedures from PA diagnosis	Median (range)	5 (1–12)	
No. of neurosurgical procedures from PC diagnosis	Median (range)	1 (0–6)	
LMD		6	30
OS from PA diagnosis (years)	Median (95% CI)	13.7 (7.7–19.7)	
OS from PC diagnosis (years)	Median (95% CI)	8.6 (0.7–16.4)	

^α^ Where data were available. Abbreviations: ACTH: adrenocorticotropic hormone; CNS: central nervous system; CI: confidence interval; FSH: follicle-stimulating hormone; GH: growth hormone; IHC: immunohistochemical staining; LH: luteinizing hormone; LMD: leptomeningeal disease; LN: lymph nodes; PA: pituitary adenoma; PC: pituitary carcinoma; PRL: prolactin.

**Table 2:** List of all the neurosurgical procedures performed in our cohort of 20 patients.

Pt. Case No.	Total No. of Proc.	Surgery No.	Proc. Type	Indication for Surgery	EOR	Decompression of Optic Apparatus	Visual Outcome	Complications
1	9	1	Transsphenoidal resection	Primary tumor resection; double vision	ST	Yes	Double vision improved, then the patient returned with tumor recurrence, after which the patient received radiation treatment, and his vision improved	NR
		2	Pre-sigmoid petrosal approach for combined R supra- and infratentorial craniotomy	Tumor recurrence	GT			NR
		3	R sub-temporal craniotomy	Tumor recurrence	ST			NR
		4	Redo R petrosal and temporal craniotomy; combined supratentorial/infratentorial approach	Tumor recurrence	GT			Wound infection, thrombosis of sigmoid sinus
		5	Wound exploration/incision and drainage	Wound infection	-			NR
		6	Redo R middle and posterior fossa craniotomy	Tumor recurrence	ST			R CN VII palsy with corneal bacterial ulceration/exposure keratopathy; minor CSF leak requiring minor bedside over-suturing
		7	SRS	Prompt tumor recurrence	-			NR
		8	R petrosal/retro-mastoid craniotomy; EVD placement	Tumor recurrence	ST			Epidural and subdural hematoma
		9	Craniotomy	Epidural and subdural hematoma evacuation	-			NR
2	3	1	Transsphenoidal resection	Primary tumor resection, symptom control	ST			NR
		2	R temporal craniotomy	Further surgical debulking of the residual tumor	ST			Panhypopituitarism following adjuvant RT
		3	Biopsy of sinus lesion	Fever, sinusitis, found to have a sinus tumor emanating from the R sphenoid and ethmoid region	-			NR
3	5	1	Transsphenoidal resection	Primary tumor resection; visual field deficit	ST	Yes	Regained full vision back	NR
		2	Craniotomy—NOS	Further surgical debulking of the residual tumor one week later	ST	Yes		NR
		3	Craniotomy—NOS	Temporal dural metastasis resection causing increased ICP symptoms	ST			NR
		4	L fronto-orbito-zygomatic craniotomy with cavernous sinus exenteration	Tumor recurrence; no visual field deficit; diplopia with L CN 6 palsy	GT	Yes	Complete vision loss in L eye; no gross movement in L eye extraocular muscles	CSF rhinorrhea; transient DI
		5	Reopening of fronto-orbito-zygomatic craniotomy, repacking of frontal and sphenoid sinuses with autologous temporalis muscle and fibrin glue, placement of R EVD	Closure of CSF leak	-			NR
4	3	1	Transsphenoidal resection	Primary tumor resection; visual loss	ST	Yes	Improvement in vision	NR
		2	Craniotomy—NOS	Further surgical debulking of the residual tumor	NA			Hypopituitarism
		3	Complete laminectomy and facetectomy at T2 for decompression of the spinal canal (transpedicular approach), arthrodesis at T1-3 using bone graft, and pedicle screw instrumentation.	Significant epidural spinal cord compression	-			NR
5	2	1	Transsphenoidal resection	Symptom control; visual field deficit in L eye	ST	Yes	Vision improvement	Transient DI; hypopituitarism
		2	L frontotemporal craniotomy, orbital and zygomatic osteotomy, exenteration of the L frontal sinus, and resection of the tumor	Emergency; tumor recurrence; symptom control; very rapid decline in the vision of the L eye associated with acute excruciating headache and complete ophthalmoplegia	ST	Yes	Complete vision loss in L eye	Transient DI; transient expressive dysphagia
6	8	1	Transsphenoidal resection	Primary tumor resection; R visual field deficit	ST	Yes	Resolution of visual field deficits	NR
		2	Craniotomy for further tumor debulking	Further surgical debulking of the residual tumor	ST	Yes		NR
		3	T7, T10, T12, L1 kyphoplasty/vertebroplasty and T8 biopsy of spinal lesion	Metastasectomy and decompression	-			NR
		4	Sublabial transsphenoidal approach to clivus and pituitary fossa.	Tumor recurrence	ST			CSF leak
		5	Placement of autologous fat graft to the sphenoid sinus and lumbar spinal drain	Closure of CSF leak	-			NR
		6	SRS	Metastasis	-			NR
		7	Endonasal transsphenoidal resection of pituitary tumor	Tumor recurrence; obtain further biopsy material to help plan targeted therapy	ST			NR
		8	R fronto-temporal parietal craniotomy	Tumor recurrence, worsening vision on the R	ST	Yes	Complete vision loss in R eye	Hypopituitarism
7	5	1	Biopsy of sellar mass	Establishing diagnosis; R vision changes and L lateral hemianopsia; pathology read as low-grade glioma at outside hospital	-			NR
		2	Craniotomy—NOS	Worsening vision on the R; tumor progression	ST	Yes	Outcome: not available	SIADH; transient L hemiparesis; R internal capsule ischemia
		3	Transsphenoidal resection	Further surgical debulking of residual tumor	ST	Yes		NR
		4	Craniotomy—NOS	Tumor recurrence; patient already with complete blindness in R eye secondary to R central retinal artery occlusion, pale retina with multiple hemorrhages, and loud bruit; L eye hemianopsia deterioration	NA	Yes	Stable vision	NR
		5	SRS	Metastasis	-			NR
8	9	1	Transsphenoidal resection	Symptom control; diplopia	ST		Improvement in diplopia	NR
		2	SRS	Adjuvant; residual tumor	-			NR
		3	Endonasal transsphenoidal tumor resection	Tumor recurrence	ST			CSF leak
		4	Lumbar drain placement	Closure of CSF leak	-			NR
		5	R frontotemporal/pterional craniotomy	Tumor recurrence, opening of R cavernous sinus for the removal of the tumor	ST	Yes	Regained full vision back	Cranial nerve III palsy; persistent DI
		6	L pterional craniotomy	Tumor recurrence; progressive temporal visual field deficit on the R	ST	Yes	Stable partial vision loss on the R	NR
		7	Endonasal transsphenoidal resection of pituitary tumor	Tumor recurrence	ST	Yes	Partial vision loss on the L	NR
		8	R VP shunt placed via parieto-occipital bur hole	Hydrocephalus due to metastasis and LMD	-			Malposition of VP shunt
		9	Revision of R VP shunt	Misposition of VP shunt	-			NR
9	5	1	Transsphenoidal resection	Symptom control; visual field deficit	ST	Yes	Vision improvement	NR
		2	R frontotemporal craniotomy	Tumor recurrence	ST			NR
		3	L orbital frontal craniotomy with resection of tumor adjacent to the L optic nerve/invasive into the L optic nerve sheath	Tumor recurrence; patient with enucleated R eye from R orbital exenteration; progressive L eye visual loss and compression of L optic nerve	NA	Yes	Stable L eye vision subjectively, but improved objectively on physical exam	NR
		4	SRS	Metastasis	-			NR
		5	R frontotemporal craniotomy for excision of cyst and radiation necrosis	Tumor recurrence; history of orbital exenteration on the R secondary to tumor invasion; compression of L optic nerve with progression to near blindness in L eye	ST	Yes	Improvement to blurry vision in L eye	NR
10	4	1	Transsphenoidal resection	Primary tumor resection; prolactinoma resistant to dopaminergic agonist	NA			NR
		2	L frontotemporal craniotomy	Tumor recurrence	NA			NR
		3	Redo L frontal craniotomy	Tumor recurrence around L optic nerve; Partial vision loss progressing to complete vision loss in L eye over 6 months	ST	Yes	Complete vision loss in L eye with no improvement, progressive partial vision loss in R eye	Seizures; altered mental status
		4	SRS	Metastasis	-			NR
11	1	1	Transsphenoidal resection	Primary tumor resection; prolactinoma resistant to dopaminergic agonist	NA			NR
12	5	1	Craniotomy—NOS	Primary tumor resection, symptom control	ST			Panhypopituitarism; persistent DI
		2	Transsphenoidal resection	Tumor recurrence	ST			CSF leak
		3	Lumbar drain placement	CSF leak	-			NR
		4	SRS	Adjuvant; residual tumor	-			NR
		5	R frontotemporal craniotomy	Metastasis diagnosis; resection of dural lesions	GT			Wound infection
13	4	1	Transsphenoidal resection	Primary tumor resection, bitemporal visual deficits	NA	Yes	Resolution of visual field deficits	NR
		2	SRS	Tumor recurrence	-			NR
		3	Trans-facial (R lateral rhinotomy) approach to tumor	Tumor recurrence and extracranial/skull base extension	ST			NR
		4	R frontotemporal craniotomy and sub-temporal craniectomy; division of R trigeminal nerve (V3)	Tumor recurrence in the middle cranial fossa and wrapping around V3 division of the cranial nerve V	NA			Oropharyngeal dysphagia
14	2	1	Transsphenoidal resection	Primary tumor resection, symptom control	ST			NR
		2	R pterional craniotomy	Further surgical debulking of the residual tumor	NA			R CN III palsy
15	7	1	Transsphenoidal resection	Pituitary apoplexy	NA			L CN VI injury
		2	Transsphenoidal resection	Tumor recurrence	NA			NR
		3	Craniotomy—NOS	Tumor recurrence involving the R cavernous sinus	ST			R CN III, IV, VI injury, anosmia
		4	SRS	Adjuvant; residual tumor	-			NR
		5	R retro-sigmoid craniotomy	Tumor recurrence involving the posterior fossa, Meckel’s cave, bilateral cavernous sinus	NA			NR
		6	Redo bifrontal and R temporal craniotomy with extended R orbital osteotomy	Tumor recurrence extending to the petrous apex, adjacent to the brainstem, and involving the R cavernous sinus with R ophthalmoplegia	NA			NR
		7	Transsphenoidal resection	Tumor recurrence involving the L cavernous sinus	NA			NR
16	5	1	Transsphenoidal resection	NA	NA			NA
		2	Surgical resection of tumor—NOS	NA	NA			NA
		3	Surgical resection of tumor—NOS	NA	NA			NA
		4	Surgical resection of tumor—NOS	NA	NA			NA
		5	Craniotomy—NOS	Resection of a metastatic temporal lesion	NA			NA
17	4	1	Transsphenoidal resection	Primary tumor resection, symptom control; diplopia	ST		Improvement in diplopia	NR
		2	R frontal temporal craniotomy	Further surgical debulking of the residual tumor	ST			NR
		3	Expanded endoscopic endonasal approach to cavernous sinus and middle cranial fossa	Further surgical debulking of residual tumor involving the cavernous sinus, carotid artery, cranial nerves, middle cranial fossa, and sella	ST			Transient DI
		4	SRS	Tumor recurrence	-			NR
18	12	1	Transsphenoidal resection	Visual field deficit	NA	Yes	Improved visual acuity	NR
		2	Transsphenoidal resection	Tumor recurrence; bitemporal hemianopsia	NA	Yes	Improved visual acuity	NR
		3	Craniotomy—NOS	Tumor recurrence	NA			CSF leak
		4	Craniotomy—NOS	Closure of CSF leak	-			NR
		5	Craniotomy—NOS	Tumor recurrence	NA			Transient DI
		6	R modified orbito-zygomatic craniotomy	Tumor recurrence; Loss of vision in L eye and poor vision in R eye	ST	Yes	Stable vision in R eye	CSF leak
		7	Duraplasty; Placement of R frontal external ventricular drain	Persistent postoperative subgaleal pseudo-meningocele	-			CN palsy with exposure keratopathy; altered mental status; AKI; CSF leak and fronto-temporal meningocele; hypopituitarism
		8	Conversion of ventriculostomy drain to R VP shunt	Hydrocephalus due to the sellar tumor-associated cyst blocking the foramen of Monroe on each side	-			VP shunt malfunction; DVT/PE
		9	Placement of a new L VP shunt, revision of R VP shunt, and fenestration of the tumor-associated cyst	Hydrocephalus from shunt malfunction	-			VP shunt malfunction and infection
		10	Ventriculostomy placement	Altered mental status, seizure, patient not responsive	-			NR
		11	Removal of VP shunts, replacement of L EVD	Infection of the shunt system	-			NR
		12	Creation of R VP shunt, endoscopic fenestration of septum pellucidum, and removal of EVD	Infection cleared	-			NR
19	2	1	L frontal craniotomy	Symptom control	ST			NA
		2	Laminectomy at T5–T6	Metastasectomy and decompression	-			NA
20	7	1	Transsphenoidal resection	Symptom control; L eye visual field defect	ST	Yes	Complete vision loss in L eye	NR
		2	SRS	Adjuvant; residual tumor	-			NR
		3	Transsphenoidal resection	Tumor recurrence	ST			NR
		4	Combined transsphenoidal with bifrontal craniotomy	Tumor recurrence	GT			CSF leak
		5	SRS	Metastasis	-			NR
		6	R pterional/sub-frontal craniotomy	Tumor recurrence; complete loss of vision in L eye and incomplete loss of vision in R eye	ST	Yes	Patient currently being followed	Multi-compartmental hemorrhage
		7	Redo L supratentorial frontal craniotomy	Evacuation of subgaleal, epidural, and subdural hematoma	-			NR

Highlighted in green are procedures done at or after the diagnosis of metastatic disease. The average # of surgeries/year pre-PC diagnosis was 0.6/year, and the average # of surgeries/year post-PC diagnosis was 0.9/year. Abbreviations: AKI, acute kidney injury; CN, cranial nerve; CSF, cerebrospinal fluid; DI, diabetes insipidus; GT, gross total; L, left; NA, not available; NOS, not otherwise specified; NR, none reported; Pt, patient; proc, procedure; R, right; RT, radiation therapy; SIADH, syndrome of inappropriate antidiuretic hormone release; SRS, stereotactic radiosurgery; ST, subtotal; VP, ventriculoperitoneal.

**Table 3:** Postoperative complications.

Variable	*N* = 45 (%)
CSF leak	9 (20)
DI	7 (16)
Permanent DI	2 (4)
Transient DI	5 (11)
Hypo- or panhypopituitarism	6 (13)
CN palsy ± exposure keratopathy	5 (11)
VP shunt malfunction, malposition, and infection	3 (7)
Intracranial hemorrhage (EDH, SDH, subgaleal hemorrhage)	2 (4)
Transient dysphagia	2 (4)
Wound infection	2 (4)
Acute kidney injury	1 (2)
Altered mental status	1 (2)
DVT/PE	1 (2)
Internal capsule ischemia	1 (2)
Seizure	1 (2)
SIADH	1 (2)
Thrombosis of the sigmoid sinus	1 (2)
Weakness	1 (2)

Abbreviations: CN, cranial nerve; DI, diabetes insipidus; DVT, deep vein thrombosis; EDH, epidural hemorrhage; PE, pulmonary embolism; SDH, subdural hemorrhage; SIADH, syndrome of inappropriate antidiuretic hormone release; VP, ventriculoperitoneal.

**Table 4:** Odds of surgical complications among repeat procedures (excluding stereotactic radiosurgery). Boldface indicates statistical significance.

Procedure	No. of Patients with Complications	No. of Patients Without Complication	Total	Rate (%)	Odds Ratio	*p*-Value
1	3	15	18	16.7	*ref*	
2	7	10	17	41.2	3.50	0.330
3	6	9	15	40.0	3.33	0.400
≥4	8	3	11	72.7	14.29	**0.012**

## Data Availability

The data presented in this study are available on request from the corresponding author due to ethical reasons.

## Supplementary Materials

The following supporting information can be downloaded at: https://www.mdpi.com/article/10.3390/cancers18132064/s1. Figure S1. Kaplan–Meier plots of metastasis-free survival showing the relationship of the interval between PA and PC diagnosis with various predictors. Metastasis-free survival curves by (A) functional tumor status, (B) MIB-1 labeling index, and (C) the presence of locoregional invasion at PA diagnosis. *Abbreviations:* PA: pituitary adenoma; PC: pituitary carcinoma. Figure S2. Kaplan–Meier plots for overall survival from PC diagnosis are presented by different features. OS curve by (A) age at PC diagnosis, (B) MIB-1 labeling index, (C) metastasis location, (D) radiotherapy following PC diagnosis, and (E) TMZ-based therapy following PC diagnosis. *Abbreviations:* CNS: central nervous system; OS: overall survival; PC: pituitary carcinoma; TMZ: temozolomide. Figure S3. Scatterplot illustrating the relationship between the total number of neurosurgical procedures per patient and overall survival (OS) from the time of pituitary adenoma (PA) diagnosis.

## References

[1] Figueiredo E.G., Paiva W.S., Teixeira M.J. (2009). Extremely late development of pituitary carcinoma after surgery and radiotherapy. J. Neuro-Oncol..

[2] Sav A., Rotondo F., Syro L.V., Di Ieva A., Cusimano M.D., Kovacs K. (2015). Invasive, atypical and aggressive pituitary adenomas and carcinomas. Endocrinol. Metab. Clin. N. Am..

[3] McCormack A., Dekkers O.M., Petersenn S., Popovic V., Trouillas J., Raverot G., Burman P., ESE Survey Collaborators (2018). Treatment of aggressive pituitary tumours and carcinomas: Results of a European Society of Endocrinology (ESE) survey 2016. Eur. J. Endocrinol..

[4] Burman P., Trouillas J., Losa M., McCormack A., Petersenn S., Popovic V., Theodoropoulou M., Raverot G., Dekkers O.M., ESE Survey Collaborators (2022). Aggressive pituitary tumours and carcinomas, characteristics and management of 171 patients. Eur. J. Endocrinol..

[5] Burman P., Casar-Borota O., Perez-Rivas L.G., Dekkers O.M. (2023). Aggressive pituitary tumors and pituitary carcinomas: From pathology to treatment. J. Clin. Endocrinol. Metab..

[6] De Sousa S.M.C., McCormack A.I., Feingold K.R., Anawalt B., Blackman M.R., Boyce A., Chrousos G., Corpas E., de Herder W.W., Dhatariya K., Dungan K., Hofland J. (2000). Aggressive pituitary tumors and pituitary carcinomas. Endotext.

[7] Raverot G., Ilie M.D., Lasolle H., Amodru V., Trouillas J., Castinetti F., Brue T. (2021). Aggressive pituitary tumours and pituitary carcinomas. Nat. Rev. Endocrinol..

[8] Raymond P., Raverot G., Ilie M.D. (2023). Outcome and prognostic factors for pituitary carcinomas: Lessons from a systematic review. Endocr.-Relat. Cancer.

[9] Asa S.L., Mete O., Perry A., Osamura R.Y. (2022). Overview of the 2022 WHO classification of pituitary tumors. Endocr. Pathol..

[10] Guastamacchia E., Triggiani V., Tafaro E., De Tommasi A., De Tommasi C., Luzzi S., Sabba C., Resta F., Terreni M.R., Losa M. (2007). Evolution of a prolactin-secreting pituitary microadenoma into a fatal carcinoma: A case report. Minerva Endocrinol..

[11] Koyama J., Ikeda K., Shose Y., Kimura M., Obora Y., Kohmura E. (2007). Long-term survival with non-functioning pituitary carcinoma-case report. Neurol. Med. Chir..

[12] Miermeister C.P., Petersenn S., Buchfelder M., Fahlbusch R., Ludecke D.K., Holsken A., Bergmann M., Knappe H.U., Hans V.H., Flitsch J. (2015). Histological criteria for atypical pituitary adenomas-data from the German pituitary adenoma registry suggests modifications. Acta Neuropathol. Commun..

[13] Pasquel F.J., Vincentelli C., Brat D.J., Oyesiku N.M., Ioachimescu A.G. (2013). Pituitary carcinoma in situ. Endocr. Pract..

[14] Kaltsas G.A., Nomikos P., Kontogeorgos G., Buchfelder M., Grossman A.B. (2005). Clinical review: Diagnosis and management of pituitary carcinomas. J. Clin. Endocrinol. Metab..

[15] Dudziak K., Honegger J., Bornemann A., Horger M., Mussig K. (2011). Pituitary carcinoma with malignant growth from first presentation and fulminant clinical course—Case report and review of the literature. J. Clin. Endocrinol. Metab..

[16] Lopes M.B., Scheithauer B.W., Schiff D. (2005). Pituitary carcinoma: Diagnosis and treatment. Endocrine.

[17] Garrao A.F., Sobrinho L.G., Pedro O., Bugalho M.J., Boavida J.M., Raposo J.F., Loureiro M., Limbert E., Costa I., Antunes J.L. (1997). ACTH-producing carcinoma of the pituitary with haematogenic metastases. Eur. J. Endocrinol..

[18] Heaney A.P. (2011). Clinical review: Pituitary carcinoma: Difficult diagnosis and treatment. J. Clin. Endocrinol. Metab..

[19] Morokuma H., Ando T., Hayashida T., Horie I., Inoshita N., Murata F., Ueki I., Nakamura K., Imaizumi M., Usa T. (2012). A Case of Nonfunctioning Pituitary Carcinoma that Responded to Temozolomide Treatment. Case Rep. Endocrinol..

[20] Kamiya-Matsuoka C., Cachia D., Waguespack S.G., Crane C.H., Mahajan A., Brown P.D., Nam J.Y., McCutcheon I.E., Penas-Prado M. (2016). Radiotherapy with concurrent temozolomide for the management of extraneural metastases in pituitary carcinoma. Pituitary.

[21] Pernicone P.J., Scheithauer B.W., Sebo T.J., Kovacs K.T., Horvath E., Young W.F., Lloyd R.V., Davis D.H., Guthrie B.L., Schoene W.C. (1997). Pituitary carcinoma: A clinicopathologic study of 15 cases. Cancer.

[22] Du Four S., Van Der Veken J., Duerinck J., Vermeulen E., Andreescu C.E., Bruneau M., Neyns B., Velthoven V., Velkeniers B. (2022). Pituitary carcinoma-case series and review of the literature. Front. Endocrinol..

[23] Raverot G., Burman P., Abreu A.P., Heaney A.P., van Hulsteijn L., Lin A.L., Marcus H., Mccormack A., Minniti G., Petersenn S. (2025). Revised European Society of Endocrinology Clinical Practice Guideline for the management of aggressive pituitary tumours and pituitary carcinomas. Eur. J. Endocrinol..

[24] Borba C.G., Batista R.L., Musolino N.R., Machado V.C., Alcantara A.E., da Silva G.O., Sperling Cescato V.A., da Cunha Neto M.B. (2015). Progression of an invasive ACTH pituitary macroadenoma with cushing’s disease to pituitary carcinoma. Case Rep. Oncol. Med..

[25] Salehi F., Agur A., Scheithauer B.W., Kovacs K., Lloyd R.V., Cusimano M. (2009). Ki-67 in pituitary neoplasms: A review—Part I. Neurosurgery.

[26] Turner H.E., Wass J.A. (1999). Are markers of proliferation valuable in the histological assessment of pituitary tumours?. Pituitary.

[27] Di Ieva A., Rotondo F., Syro L.V., Cusimano M.D., Kovacs K. (2014). Aggressive pituitary adenomas—Diagnosis and emerging treatments. Nat. Rev. Endocrinol..

[28] Zaidi H.A., Cote D.J., Dunn I.F., Laws E.R. (2016). Predictors of aggressive clinical phenotype among immunohistochemically confirmed atypical adenomas. J. Clin. Neurosci..

[29] Gurlek A., Karavitaki N., Ansorge O., Wass J.A. (2007). What are the markers of aggressiveness in prolactinomas? Changes in cell biology, extracellular matrix components, angiogenesis and genetics. Eur. J. Endocrinol..

[30] Brown R.L., Wollman R., Weiss R.E. (2007). Transformation of a pituitary macroadenoma into a corticotropin-secreting carcinoma over 16 years. Endocr. Pract..

[31] Ragel B.T., Couldwell W.T. (2004). Pituitary carcinoma: A review of the literature. Neurosurg. Focus.

[32] Raverot G., Burman P., McCormack A., Heaney A., Petersenn S., Popovic V., Trouillas J., Dekkers O.M., European Society of Endocrinology (2018). European Society of Endocrinology Clinical Practice Guidelines for the management of aggressive pituitary tumours and carcinomas. Eur. J. Endocrinol..

[33] Ji Y., Vogel R.I., Lou E. (2016). Temozolomide treatment of pituitary carcinomas and atypical adenomas: Systematic review of case reports. Neurooncol. Pract..

[34] Jouanneau E., Wierinckx A., Ducray F., Favrel V., Borson-Chazot F., Honnorat J., Trouillas J., Raverot G. (2012). New targeted therapies in pituitary carcinoma resistant to temozolomide. Pituitary.

[35] Losa M., Bogazzi F., Cannavo S., Ceccato F., Curto L., De Marinis L., Iacovazzo D., Lombardi G., Mantovani G., Mazza E. (2016). Temozolomide therapy in patients with aggressive pituitary adenomas or carcinomas. J. Neurooncol..

[36] Ortiz L.D., Syro L.V., Scheithauer B.W., Ersen A., Uribe H., Fadul C.E., Rotondo F., Horvath E., Kovacs K. (2012). Anti-VEGF therapy in pituitary carcinoma. Pituitary.

[37] Scheithauer B.W., Kurtkaya-Yapicier O., Kovacs K.T., Young W.F., Lloyd R.V. (2005). Pituitary carcinoma: A clinicopathological review. Neurosurgery.

[38] Verma J., McCutcheon I.E., Waguespack S.G., Mahajan A. (2014). Feasibility and outcome of re-irradiation in the treatment of multiply recurrent pituitary adenomas. Pituitary.

[39] Santos-Pinheiro F., Penas-Prado M., Kamiya-Matsuoka C., Waguespack S.G., Mahajan A., Brown P.D., Shah K.B., Fuller G.N., McCutcheon I.E. (2019). Treatment and long-term outcomes in pituitary carcinoma: A cohort study. Eur. J. Endocrinol..

[40] Majd N., Waguespack S.G., Janku F., Fu S., Penas-Prado M., Xu M., Alshawa A., Kamiya-Matsuoka C., Raza S.M., McCutcheon I.E. (2020). Efficacy of pembrolizumab in patients with pituitary carcinoma: Report of four cases from a phase II study. J. Immunother. Cancer.

[41] Yang Y.X., Liang W.L., Fan K.X., Yang T., Cheng J.M. (2024). Clinical features of pituitary carcinoma: Analysis based on a case report and literature review. Front. Endocrinol..

[42] Yearley A.G., Chalif E.J., Gupta S., Chalif J.I., Bernstock J.D., Nawabi N., Arnaout O., Smith T.R., Reardon D.A., Laws E.R. (2023). Metastatic pituitary tumors: An institutional case series. Pituitary.

[43] Chatzellis E., Alexandraki K.I., Androulakis I.I., Kaltsas G. (2015). Aggressive pituitary tumors. Neuroendocrinology.

[44] Heaney A.P., Melmed S. (2004). Molecular targets in pituitary tumours. Nat. Rev. Cancer.

[45] Kasuki L., Raverot G. (2020). Definition and diagnosis of aggressive pituitary tumors. Rev. Endocr. Metab. Disord..

[46] Rutkowski M.J., Alward R.M., Chen R., Wagner J., Jahangiri A., Southwell D.G., Kunwar S., Blevins L., Lee H., Aghi M.K. (2018). Atypical pituitary adenoma: A clinicopathologic case series. J. Neurosurg..

[47] Shao S., Li X. (2013). Clinical features and analysis in 1385 Chinese patients with pituitary adenomas. J. Neurosurg. Sci..

[48] Cohen A.C., Goldney D.C., Danilowicz K., Manavela M., Rossi M.A., Gomez R.M., Cross G.E., Bruno O.D. (2019). Long-term outcome after bilateral adrenalectomy in Cushing’s disease with focus on Nelson’s syndrome. Arch. Endocrinol. Metab..

[49] Ritzel K., Beuschlein F., Mickisch A., Osswald A., Schneider H.J., Schopohl J., Reincke M. (2013). Clinical review: Outcome of bilateral adrenalectomy in Cushing’s syndrome: A systematic review. J. Clin. Endocrinol. Metab..

[50] Thapar K., Kovacs K., Scheithauer B.W., Stefaneanu L., Horvath E., Pernicone P.J., Murray D., Laws E.R. (1996). Proliferative activity and invasiveness among pituitary adenomas and carcinomas: An analysis using the MIB-1 antibody. Neurosurgery.

[51] Del Basso De Caro M., Solari D., Pagliuca F., Villa A., Guadagno E., Cavallo L.M., Colao A., Pettinato G., Cappabianca P. (2017). Atypical pituitary adenomas: Clinical characteristics and role of ki-67 and p53 in prognostic and therapeutic evaluation. A series of 50 patients. Neurosurg. Rev..

[52] Chiloiro S., Doglietto F., Trapasso B., Iacovazzo D., Giampietro A., Di Nardo F., de Waure C., Lauriola L., Mangiola A., Anile C. (2015). Typical and atypical pituitary adenomas: A single-center analysis of outcome and prognosis. Neuroendocrinology.

[53] de Aguiar P.H., Aires R., Laws E.R., Isolan G.R., Logullo A., Patil C., Katznelson L. (2010). Labeling index in pituitary adenomas evaluated by means of MIB-1: Is there a prognostic role? A critical review. Neurol. Res..

[54] Tanizaki Y., Jin L., Scheithauer B.W., Kovacs K., Roncaroli F., Lloyd R.V. (2007). P53 gene mutations in pituitary carcinomas. Endocr. Pathol..

[55] Casar-Borota O., Boldt H.B., Engstrom B.E., Andersen M.S., Baussart B., Bengtsson D., Berinder K., Ekman B., Feldt-Rasmussen U., Hoybye C. (2021). Corticotroph aggressive pituitary tumors and carcinomas frequently harbor atrx mutations. J. Clin. Endocrinol. Metab..

[56] Sun F., Grenert J.P., Tan L., Van Ziffle J., Joseph N.M., Mulvey C.K., Bergsland E. (2022). Checkpoint inhibitor immunotherapy to treat temozolomide-associated hypermutation in advanced atypical carcinoid tumor of the lung. JCO Precis Oncol..

[57] Klempner S.J., Hendifar A., Waters K.M., Nissen N., Vail E., Tuli R., Mita A. (2020). Exploiting temozolomide-induced hypermutation with pembrolizumab in a refractory high-grade neuroendocrine neoplasm: A proof-of-concept case. JCO Precis Oncol..

[58] Priola S.M., Esposito F., Cannavo S., Conti A., Abbritti R.V., Barresi V., Baldari S., Ferrau F., Germano A., Tomasello F. (2017). Aggressive pituitary adenomas: The dark side of the moon. World Neurosurg..

[59] Chang E.F., Sughrue M.E., Zada G., Wilson C.B., Blevins L.S., Kunwar S. (2010). Long term outcome following repeat transsphenoidal surgery for recurrent endocrine-inactive pituitary adenomas. Pituitary.

[60] Benveniste R.J., King W.A., Walsh J., Lee J.S., Delman B.N., Post K.D. (2005). Repeated transsphenoidal surgery to treat recurrent or residual pituitary adenoma. J. Neurosurg..

[61] Patil C.G., Veeravagu A., Prevedello D.M., Katznelson L., Vance M.L., Laws E.R. (2008). Outcomes after repeat transsphenoidal surgery for recurrent Cushing’s disease. Neurosurgery.

[62] Shimon I., Jallad R.S., Fleseriu M., Yedinak C.G., Greenman Y., Bronstein M.D. (2015). Giant GH-secreting pituitary adenomas: Management of rare and aggressive pituitary tumors. Eur. J. Endocrinol..

[63] Shimon I., Sosa E., Mendoza V., Greenman Y., Tirosh A., Espinosa E., Popovic V., Glezer A., Bronstein M.D., Mercado M. (2016). Giant prolactinomas larger than 60 mm in size: A cohort of massive and aggressive prolactin-secreting pituitary adenomas. Pituitary.

[64] Heaney A. (2014). Management of aggressive pituitary adenomas and pituitary carcinomas. J. Neuro-Oncol..

[65] Buchfelder M. (2009). Management of aggressive pituitary adenomas: Current treatment strategies. Pituitary.

[66] Bakhsheshian J., Wheeler S., Strickland B.A., Pham M.H., Rennert R.C., Carmichael J., Weiss M., Zada G. (2019). Surgical outcomes following repeat transsphenoidal surgery for nonfunctional pituitary adenomas: A retrospective comparative study. Oper. Neurosurg..

[67] Graillon T., Castinetti F., Fuentes S., Gras R., Brue T., Dufour H. (2020). Transcranial approach in giant pituitary adenomas: Results and outcome in a modern series. J. Neurosurg. Sci..

[68] Raverot G., Castinetti F., Jouanneau E., Morange I., Figarella-Branger D., Dufour H., Trouillas J., Brue T. (2012). Pituitary carcinomas and aggressive pituitary tumours: Merits and pitfalls of temozolomide treatment. Clin. Endocrinol..

[69] Park K.S., Hwang J.H., Hwang S.K., Kim S., Park S.H. (2014). Pituitary carcinoma with fourth ventricle metastasis: Treatment by excision and Gamma-knife radiosurgery. Pituitary.

[70] Joehlin-Price A.S., Hardesty D.A., Arnold C.A., Kirschner L.S., Prevedello D.M., Lehman N.L. (2017). Case report: ACTH-secreting pituitary carcinoma metastatic to the liver in a patient with a history of atypical pituitary adenoma and Cushing’s disease. Diagn. Pathol..

[71] Ayuk J., Natarajan G., Geh J.I., Mitchell R.D., Gittoes N.J. (2005). Pituitary carcinoma with a single metastasis causing cervical spinal cord compression. Case report. J. Neurosurg. Spine.

[72] Lasolle H., Cortet C., Castinetti F., Cloix L., Caron P., Delemer B., Desailloud R., Jublanc C., Lebrun-Frenay C., Sadoul J.L. (2017). Temozolomide treatment can improve overall survival in aggressive pituitary tumors and pituitary carcinomas. Eur. J. Endocrinol..

