# Supplementary material for: Role of Surgery in the Multimodal Treatment of Pituitary Carcinoma: A Retrospective Single-Institution Case Series
Source: Cancers (Basel). 2026 Jun 25;18(13):2064. doi: 10.3390/cancers18132064 (PMC13359615; doi:10.3390/cancers18132064)
Supplement: Supplementary file 1 [file cancers-18-02064-s001.zip › cancers-4315607-supplementary.pdf]

cancers

Surgery in multimodal treatment of pituitary carcinoma: a single-institution experience

Supplementary Information

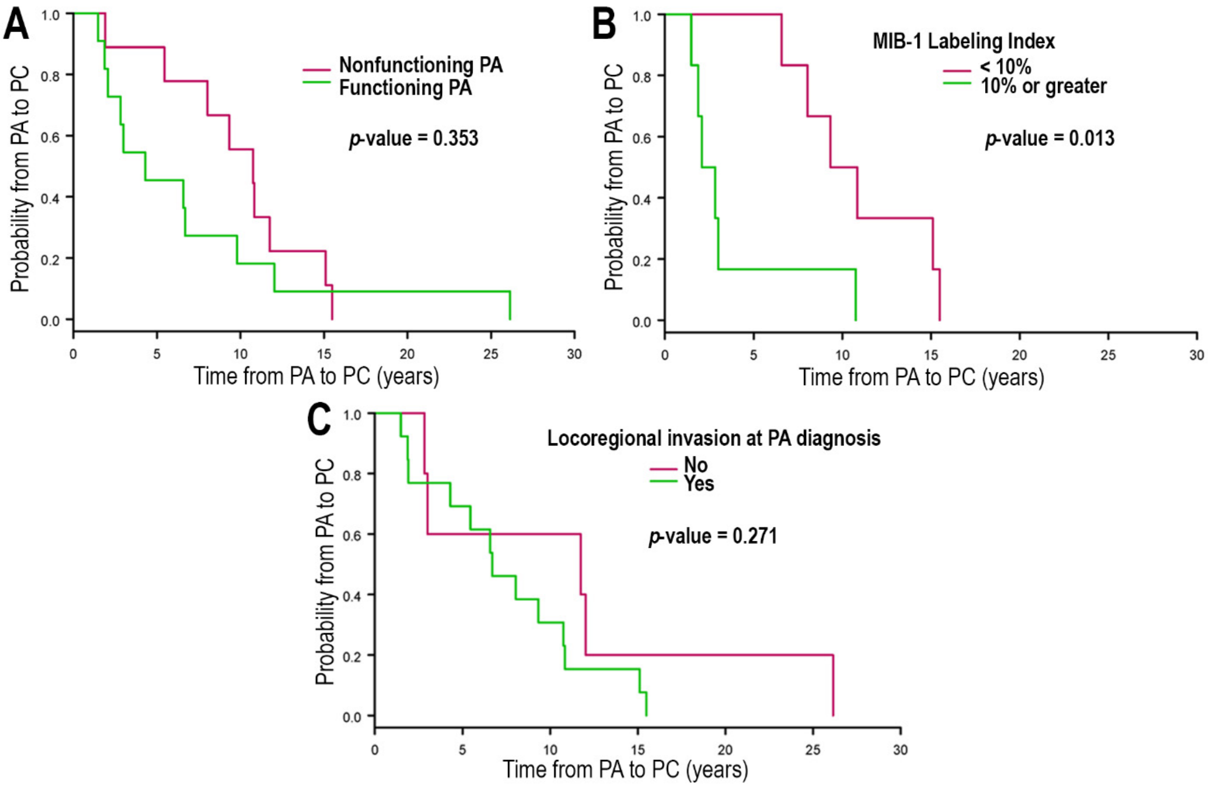

Figure S1. Kaplan-Meier plots for metastasis-free survival showing the relationship of the interval between PA and PC diagnosis with various predictors. Metastasis-free survival curves by **(A)** functional tumor status, **(B)** MIB-1 labeling index, and **(C)** the presence of locoregional invasion at PA diagnosis. *Abbreviations:* PA: pituitary adenoma; PC: pituitary carcinoma.

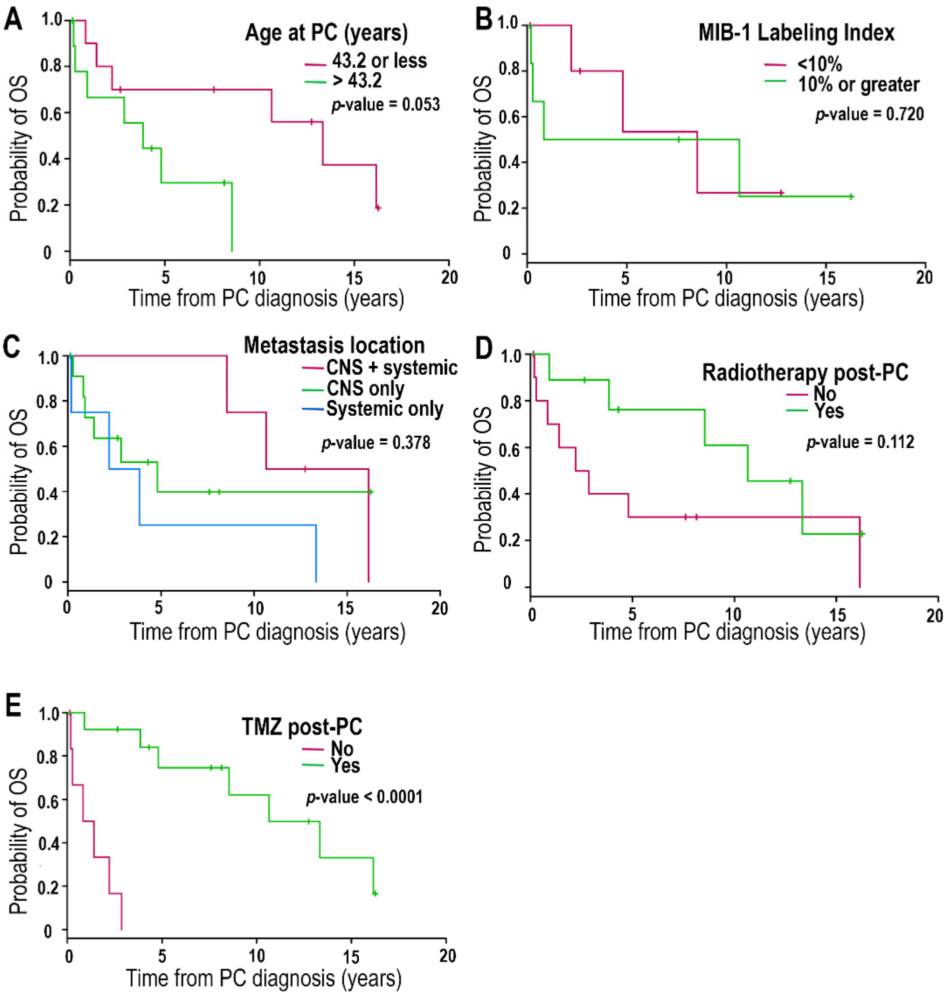

Figure S2. Kaplan-Meier plots for overall survival from PC diagnosis are presented by different features. OS curve by **(A)** age at PC diagnosis, **(B)** MIB-1 labeling index, **(C)** metastasis location, **(D)** radiotherapy following PC diagnosis, and **(E)** TMZ-based therapy following PC diagnosis. *Abbreviations:* CNS: central nervous system; OS: overall survival; PC: pituitary carcinoma; TMZ: temozolomide.

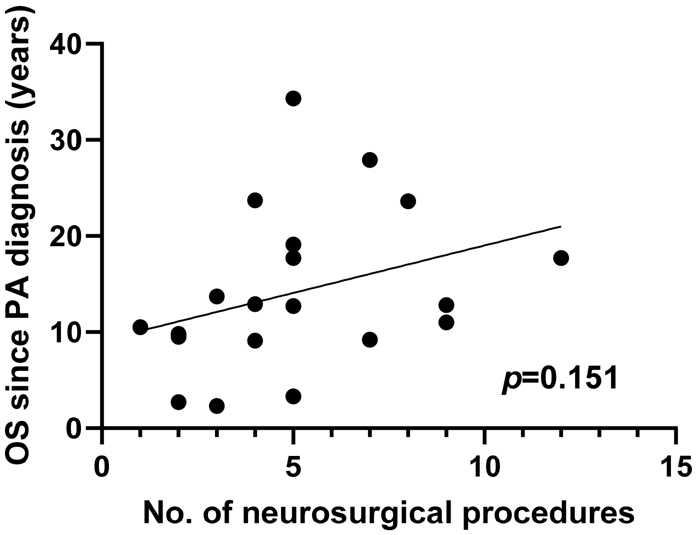

Figure S3. Scatterplot illustrating the relationship between the total number of neurosurgical procedures per patient and overall survival (OS) from the time of pituitary adenoma (PA) diagnosis.
